# Development of Electrochemical Nanosensor for the Detection of Malaria Parasite in Clinical Samples

**DOI:** 10.3389/fchem.2019.00089

**Published:** 2019-02-25

**Authors:** Olaoluwa R. Obisesan, Abolanle S. Adekunle, John A. O. Oyekunle, Thomas Sabu, Thabo T. I. Nkambule, Bhekie B. Mamba

**Affiliations:** ^1^Department of Chemistry, Obafemi Awolowo University, Ile-Ife, Nigeria; ^2^International and Inter University Center for Nanoscience and Nanotechnology, Mahatma Gandhi University, Kottayam, India; ^3^Nanotechnology and Water Sustainability Research Unit, College of Science, Engineering and Technology, Univeristy of South Africa, Johannesburg, South Africa

**Keywords:** β-hematin, metal oxide nanoparticles, sensor, cyclic voltammetry, square wave voltammetry, malaria, clinical samples, biomarker

## Abstract

In this study, electrochemical nanosensors were developed from the synthesized metal oxide (MO) nanoparticles by supporting it on a gold electrode (Au). The activity of the developed nanosensor toward the detection of malaria biomarker (β-hematin) was determined and the optimum conditions at which the maximum detection and quantification occurred were established. β-Hematin current response at the sensors was higher when compared with the bare Au electrode and followed the order Au-CuO (C) > Au-CuO (M) > Au-Fe_2_O3 (M) > Au-Fe_2_O3 (C) > Au-Al_2_O3 (M) > Au-Al_2_O3 (C) > bare Au. The developed sensors were stable with a relatively low current drop (10.61–17.35 %) in the analyte. Au-CuO sensor had the best performance toward the biomarker and quantitatively detected *P. berghei* in infected mice's serum samples at 3.60–4.8 mM and *P. falciparum* in human blood serum samples at 0.65–1.35 mM concentration.

## Introduction

Medical diagnosis and biological monitoring of diseases in the clinical laboratory are important because they're useful in detecting various diseases or onset of several diseases. The use of markers (biomarkers) has been the main cornerstone in the identification and quantification of target metabolites in biological fluids (blood, urine, and saliva) (Gil and Pla, 2001; Angerer et al., 2006). Detection of most diseases using sophisticated techniques mainly involves expensive and time-consuming processes. Development of sensitive, selective, accurate, rapid as well as affordable techniques for clinical diagnosis has been a major focus of researchers recently. Electrochemical sensors using nanoparticles have emerged as one of the suitable technologies for the detection of analytes of interest in clinical chemistry due to their high sensitivity and selectivity, rapid response time, and low cost (Wang, 1994).

Malaria is an intricate, infectious, hematologic disease instigated by a protozoan parasite named *Plasmodium falciparum* (Priyamvada et al., 2014). The parasite spends part of its life in humans and part in Anopheles mosquitoes. Malaria is transmitted to humans through infected Anopheles mosquitos. Although there are more than 50 species of *Plasmodium*, only four species, *P. falciparum, P. vivax, P. malariae*, and *P. ovale*, are known to cause malaria in humans (Priyamvada et al., 2014). The malaria parasite depends on iron for growth because many enzymes of the parasite metabolic pathways depend on iron (Weinberg and Moon, 2009). Malaria clinical symptoms occur during the intra-erythrocytic stage. At this stage, *P. falciparum* degrades more than 80% of hemoglobin present in the food vacuoles to serve as a source of amino acids (Goyal et al., 2012). To illustrate this phenomenon, in an average patient with 750 g of healthy hemoglobin and a 20% parasitemia, as much as 100 g of hemoglobin can be degraded during this cycle (Ziegler et al., 2001). This hemoglobin degradation results in the release of free heme or iron protoporphyrin IX (FePPIX) along with oxygen. Since this free heme is toxic to the parasite, *P. falciparum* converts the reactive heme species into a compact, highly insoluble, weakly magnetic crystal known as hemozoin with decreased pro-oxidant capacity which is nontoxic to the parasite (Oliveira et al., 2005).

Hemozoin, also known as malaria pigment, is a visible marker chemically and structurally similar to a distinctive hematin pigment, called beta-hematin (Fitch and Kanjananggulpan, 1987). Beta-hematin has been extensively explored for the synthesis of antimalaria drugs and can also serve as a biomarker for the diagnosis of malaria. It is a microcrystalline cyclic dimer of ferriprotoporphyrin IX (Fe(III)PPIX) in which the propionate side chain of one protoporphyrin coordinates to the iron(III) center of the other. The dimers use hydrogen to bond with their neighbors via propionic acid group, forming extended chains through the macroscopic crystal (Pagola et al., 2000). Considerable studies have been done on using β-hematin as a biomarker in antimalarial drug synthesis in order to inhibit the formation of hemozoins in the host system (Kumar et al., 2007; Thomas et al., 2012), as a biomarker for diagnosis in a magnetic field-enriched surface (Yuen and Liu, 2012), and as a detection assay for malaria diagnosis (Rebelo et al., 2013). With so many studies on β-hematin, however, there is a paucity of data on electrochemical detection of β-hematin as a target toward malaria diagnosis.

The motivation for this study is based on providing a long-term solution to the challenges faced by physicians and patients with malaria diagnosis. This study, therefore, focused on the development and affirmation of an electrochemical sensor that integrates metal oxide nanoparticles as electroactive species for the diagnosis of malaria parasite with the use of a biomarker (β-hematin) as the identification medium.

## Methods

### Materials and Reagents

Materials used include magnetic stirrer, microwave, sonicator, PG-581 biologic potentiostat, 3 mm diameter gold electrode (working electrode) from CHI Instruments, USA, silver/silver chloride reference electrode (in saturated KCl, 3.0 M) and platinum counter electrode from (Bio-Logic Science Instruments, France).

All reagents and solvents used in this study were of analytical grades. They include ethanol (C_2_H_5_OH), copper chloride (CuCl_2_), copper acetate (Cu(CH_3_COO)_2_, ethylene glycol (C_2_H_6_O_2_), urea (CO(NH_2_)_2_, aluminum nitrate nonahydrate (Al(NO_3_)_3_·9H_2_O), iron (II) chloride tetrahydrate (FeCl_2_.4H_2_O), ferriprotoporphyrin IX chloride (Hemin Chloride [Cl-Fe(III)PPIX]), ammonia (NH_3_), sodium hydroxide (NaOH), acetic acid (CH_3_COOH), hydrochloric acid (HCl), potassium phosphate dibasic (K_2_HPO_4_), potassium phosphate monobasic (KH_2_PO_4_), potassium ferrocyanide trihydrate (K_4_[Fe(CN)_6_].3H_2_O), potassium hexacyanoferrate (K_3_[Fe(CN)_6_]).

### Synthesis and Characterization of Metal Oxide Nanoparticles

#### Chemical Synthesis of Copper Oxide (CuO) Nanoparticles

Copper oxide nanoparticles were synthesized using the method described by Manimaran et al. (2014). One hundred milliliters of deionised water along with 1 g of NaOH pellets were added to 0.51 g of copper chloride (CuCl_2_) in a 500 mL beaker. The mixture was heated with a magnetic stirrer for 1 h during which the color of the solution changed from blue to black. After the reaction, the mixture was cooled to room temperature. The chemical reaction can be represented as:


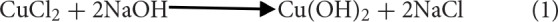



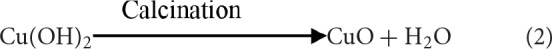


The pH value of the so formed CuO wet precipitate was neutralized by adding droplets of hydrochloric acid. Then the CuO wet precipitate was washed with deionised water to remove the impurity ions present in the solution and calcined at 300°C for 2 h. The solid is represented as CuO (C) where “C” represents chemical synthesis.

#### Microwave Synthesis of Copper Oxide (CuO) Nanoparticles

Copper acetate (Cu(CH_3_COO)_2_ and urea (CO(NH_2_))_2_ were taken as a solute in the ratio 1:3 (1 g of copper acetate and 3 g of urea) and dissolved in 100 mL ethylene glycol. The prepared solution was kept in a domestic microwave oven (operated at frequency 2.45 GHz and power 800 W). Microwave irradiation was carried out till the solvent was evaporated completely. Acetone washing was used to remove the organic impurities from the black colloidal precipitate that was obtained. The sample obtained was dried in atmospheric air and annealed for 30 min at 100°C to improve the ordering (Rejith and Krishnan, 2012). The black product is represented as CuO (M) where “M” represents microwave synthesis.

#### Chemical Synthesis of Aluminum Oxide (Al_2_O_3_) Nanoparticles

Aluminum nitrate nonahydrate (Al(NO_3_)_3_**·**9H_2_O) was used as a precursor for the synthesis of aluminum oxide (Al_2_O_3_) nanoparticles. Al(NO_3_)_3_·9H_2_O (9.38 g, 0.025 mol) was dissolved in 50 mL of distilled water to obtain a 0.5 M solution. The precursor solution obtained (8 mL) was added to a 100 mL conical flask containing a mixed solution of 50 mL distilled water, 3.5 mL NaOH (5 M), and then blended well by stirring for 15 min. Subsequently, the mixed solution was left for 5 days at room temperature. The white crystalline products formed were collected by centrifugation, washed with distilled water and ethanol several times and dried, then calcinated at 850°C for 10 h (Maryam et al., 2014). The product is represented as Al_2_O_3_ (C) where “C” represents chemical synthesis.

#### Microwave Synthesis of Aluminum Oxide (Al_2_O_3_) Nanoparticles

Microwave synthesis of aluminum oxide (Al_2_O_3_) was carried out using the method described by Maryam et al. (2014) with some modifications. Aluminum nitrate nonahydrate (Al(NO_3_)_3_·9H_2_O) was used as a precursor for the synthesis of Al_2_O_3_. Al(NO_3_)_3_·9H_2_O (9.38 g, 0.025 mol) was dissolved in 50 mL of distilled water to obtain a 0.5 M solution. The precursor solution obtained (8 mL) was added to a 100 mL conical flask containing mixed solutions of 50 mL distilled water, 3.5 mL NaOH (5 M), and then blended well by stirring for 15 min. Subsequently, the mixed solution was subjected to microwave heating at 540 W to dry it. The solid product obtained is represented as Al_2_O_3_ (M) where “M” represents microwave synthesis.

#### Chemical Synthesis of Iron Oxide (Fe_2_O_3_) Nanoparticles

The accurately prepared 0.25 M FeCl_2_·4H_2_O solution was mixed with 5.4 M NaOH solution and 1.34 M NH_3_·H_2_O solution, respectively. The resulting mixture solution was magnetically stirred and heated up to 90°C while kept under nitrogen. The duration time was 1.5 h. Meanwhile, the pH value was adjusted by dropwise addition of 0.1 M HCl solution. The color of the resulting slurry changed from grayish green to red. Then, the slurry was washed repeatedly with deionized water and the suspension of iron oxide nanoparticles was obtained (Yang et al., 2014).

#### Microwave Synthesis of Iron Oxide (Fe_2_O_3_) Nanoparticles

Accurately weighed 5.5 g of iron nitrate was mixed with 2–3 drops of ethylene glycol and placed in the microwave. The paste was boiled for a few seconds and underwent dehydration followed by decomposition with the evolution of gases (O_2_, NO_2_) with yellow colored fumes. After 30 s it began burning and releasing lots of heat with vaporization of all the solution instantly to form a foamy porous black solid powder (Virendra et al., 2013).

#### Structural and Morphological Characterization of Synthesized Nanoparticles

The synthesized nanoparticles were characterized using Ultra-violet visible (UV-visible) spectroscopy, X-ray diffraction (XRD) spectroscopy, Scanning electron microscopy (SEM).

#### Synthesis of β-Hematin

β-hematin crystals (a biomarker for malaria parasite) were synthesized using an acid-catalyzed method (Egan et al., 2001). Accurately measured 8 mL of 0.1 M NaOH solution was used to dissolve 7.9 mM of Ferriprotoporphyrin IX chloride [Cl-Fe(III)PPIX, hemin chloride], heated at 60°C and stirred at 150 rpm for 46 min. Also, 1.45 mL of HCl (1 M) and 8.825 mL of acetic acid were added to the mixture, after 10 min and 14 min, respectively. After 46 min, the mixture was left undisturbed in a dark environment for 24 h. The solute obtained was washed with methanol and deionized water sequentially, filtered and collected for drying.

(3) $$\begin{array}{r} \underset{\text{Hemin chloride}}{2\text{Cl}-\text{Fe}\left(\text{III}\right)\text{PPIX}}+\underset{\text{Acetic acid}}{{\text{CH}}_{3}\text{COOH}}\;\to \underset{\beta -\text{Hematin}}{{\left(\text{Fe}\left(\text{III}\right)\text{PPIX}\right)}_{2}}\\ +\;{\text{CH}}_{3}{\text{COOH}}^{+}{\text{Cl}}^{-} \end{array}$$

### Electrode Modification Procedure (Sensor Preparation)

Electrode modification was carried out using the drop-dry method. The surface of the working electrode (Au) was prepared by polishing in an aqueous slurry of alumina nanopowder (LabChem) on a Buehler felt pad. The electrode was put through the ultrasonic vibration for 5 min in distilled water and absolute ethanol to eliminate the remaining alumina particles that may be caught on the surface. The gold electrode was then treated with piranha solution for about 1 min and was followed by rinsing in distilled water and ethanol for another 5 min and dried.

Suspensions of metal oxide nanoparticles were prepared by dissolving 2.5, 5.0, 7.5, and 10.0 mg of each nanoparticle in 1.0 mL DMF and sonicated for 20 min. Ten (10) μL drops of the prepared suspension were dropped on the bare gold electrode and dried in an oven at 50°C for 5 min to obtain gold-metal oxide nanoparticles (Au-MO) modified electrodes. Thus, the developed sensors using this procedure include Au-CuO (C), Au-CuO (M), Au-Fe_2_O_3_ (C), Au-Fe_2_O_3_ (M), Au-Al_2_O_3_ (C), and Au-Al_2_O_3_ (M). The mass of the active material on the Au was obtained by measuring the weight of the Au electrode before and after deposition using the Mettler Toledo (Model: ML 54) analytical balance (Switzerland). The active materials were found to range between ~0.5 and 1.5 mg with the highest weight (1.5 mg) recorded for Au-CuO electrode modified with a 10 μL drop of 10 mg nanoparticle in 1 mL DMF. Thus, it could be inferred that the chemical composition of the MO nanoparticles also played a role on their adsorption and film formation on the base electrode.

### Electrochemical Studies

Electrochemical experiments were carried out to establish the successful modification of the electrodes, electron transport, and electrocatalytic properties of the bare and modified gold electrode (Au). The bare and modified Au electrode disk (*d* = 3.0 mm in Teflon) was used as the working electrode, the platinum disk as a counter electrode and Ag/AgCl, KCl (sat'd) as a reference electrode. A benchtop pH/ISE ORION meter was used for pH measurements. All solutions were de-aerated by bubbling with nitrogen prior to each electrochemical experiment. Electrochemical experiments were carried out using PG-581 biologic potentiostat driven by UiEChem Electrochemical software. CV experiments were carried out by running the bare and modified Au electrode in 0.1 M buffer solution (PBS, pH 9.0), 5 mM [Fe(CN)_6_]^4−/3−^ solution prepared in 0.1 M PBS, and 1.0 mM of β-Hematin in pH 9.0 phosphate buffer solution as supporting electrolyte.

### Interference Study

Since malaria parasite could coexist with *Salmonella typhi* bacteria in the host system, simultaneous determination of β-hematin and *S. typhi* antiserum VI was carried out. Accurately prepared 1.0 mM β-hematin in pH 9.0 PBS was spiked with 1.0–4.0 mg/mL of *Salmonella typhi* antiserum VI. The interference study of the biomarkers on Au-MO modified electrodes was carried out using a cyclic voltammetry experiment at scan rate 50 mV/s. The study was repeated in malaria-infected humans and mice sera in pH 9.0 PBS, spiked with 1.0–4.0 mg/mL *Salmonella typhi* antiserum VI and detected simultaneously with the malaria biomarker using the best Au-MO modified electrode.

### Real Sample Analysis

Prior to the collection of samples, ethical clearance certificate was obtained from the Ethics and Research Committee of Obafemi Awolowo University Health Center, Ile Ife, Nigeria. During the handling of clinical samples, disposable gloves, and laboratory coats were always used to prevent infection or transmission of diseases. All the glassware used during this experiment were cleaned with sodium hypochlorite and ethanol after each experiment.

### Recovery Analysis Using Human Urine Sample

Analytical validation of the electrochemical sensor method developed in this study was carried out using a recovery analysis experiment. Five urine samples (belonging to patients screened negative for malaria parasite) were collected at the Obafemi Awolowo University Health Centre, Ile Ife. Each urine sample was centrifuged (500 rpm) and diluted 10 times with pH 9.0 PBS. Precisely measured 10 mL of the diluted urine sample in 25 mL standard flask was spiked with 10 μM β-hematin and the content was made up to mark with pH 9.0 PBS. The spiked sample was run using SWV experiment, where Au-CuO (C) was used as the working electrode, Ag|AgCl, saturated KCl as the reference electrode, and Pt as the counter electrode, respectively. The β-hematin concentration corresponding to SWV reduction current of the spiked sample was estimated from the regression equation for the β-hematin standard calibration curve. The experiment was repeated 4 times (*n* = 4) for each urine sample and the recovery percentage was determined as the difference in the β-hematin concentration before and after the spiking experiment.

### Determination of β-Hematin in Human Serum

Human blood samples of five patients (screened positive to malaria parasite) were collected at Obafemi Awolowo University Health Center, Ile Ife, Osun State, Nigeria. The blood samples were allowed to clot and centrifuged (at 500 rpm) to separate the serum and the plasma. The sera were diluted 10 times with pH 9.0 PBS and 10 mL of each serum was analyzed for the presence of β-hematin using the standard addition method (SAM) and square wave voltammetry technique, where Au-CuO (C) was the working electrode, Ag|AgCl, sat'd KCl was reference electrode and Pt counter was the electrode, respectively. Ten milliliters of the diluted sera samples was replicated in six 25 mL standard flasks and all except one were spiked with different standard concentrations (2, 4, 6, 8, and 10 μM) of β-hematin and makeup to mark with pH 9.0 PBS. The current corresponding to each spiked sample was determined using SWV experiment. The experiment was repeated 4 times (*n* = 4) for each serum sample and β-hematin concentration in the unspiked serum was determined by extrapolation.

### Determination of β-Hematin in Animal Serum

Blood samples from 5 malaria-infected mice and 2 uninfected mice (control sample) were collected from the Faculty of Pharmacy, Obafemi Awolowo University, Ile Ife, Osun State, Nigeria. The serum was separated from the plasma by centrifugation. The sera were diluted 10 times with pH 9.0 PBS and 10 mL of each serum was analyzed for the presence of β-hematin using the standard addition method (SAM) and square wave voltammetry technique as discussed in determination of β-hematin in human serum. The experiment was repeated 4 times (*n* = 4) for each serum sample and β-hematin concentration in the unspiked serum was determined by extrapolation.

## Results and Discussion

### Microscopic Analysis

**Figure 1:**
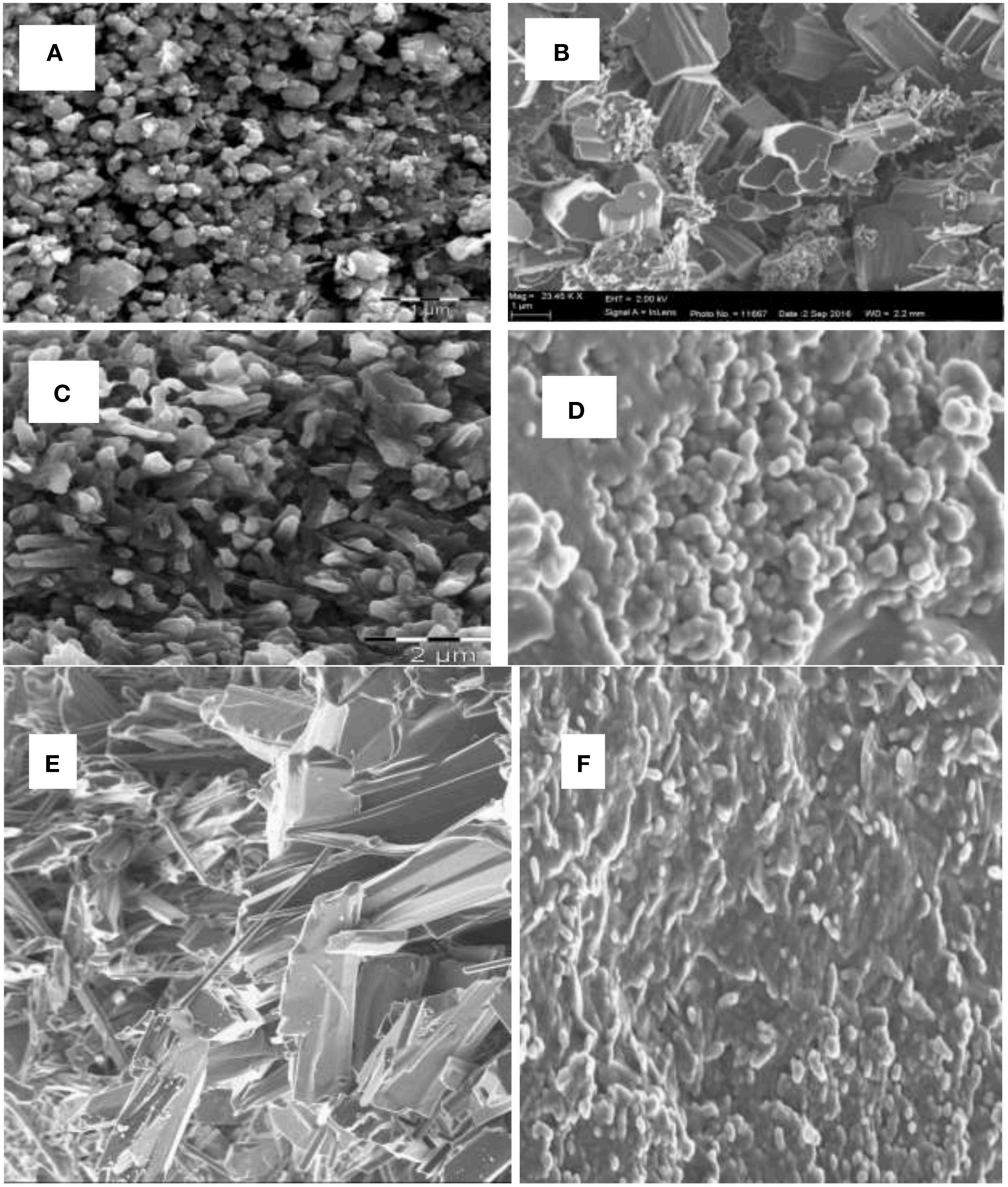
SEM image of **(A)** CuO (C) nanoparticles, **(B)** CuO (M) nanoparticles, **(C)** Al_2_O_3_ (C) nanoparticles, **(D)** Al_2_O_3_ (M) nanoparticles, **(E)** Fe_**2**_O_**3**_ (C) nanoparticles, **(F)** Fe_**2**_O_**3**_ (M) nanoparticles.

Scanning electron microscope (SEM) analysis was used for the size and morphology study of the synthesized metal oxide (MO) nanoparticles. Figures 1A–F show the SEM images of CuO, Al_2_O_3_, and Fe_2_O_3_ nanoparticles for both chemical and microwave synthesis. SEM image of CuO (C) nanoparticles showed uniformly dispersed particles that are aggregated together with an average size of 12.72 nm (Figure 1A), while CuO (M) nanoparticles showed rod-like shaped agglomerated particles with an average size of 11.16 nm (Figure 1B). The particle size of the CuO nanoparticles was lower than the 30–60 nm reported for CuO in the literature (Sutradhar et al., 2014; Dhineshbabu et al., 2016). The SEM image of Al_2_O_3_ (C) revealed a quasi-spherical shape, with the average size of the agglomerated particles of 30.37 nm (Figure 1C). The Al_2_O_3_ (M) nanoparticles showed a passivated layer with an average particle size of 20.92 nm (Figure 1D). The Al_2_O_3_ (C) particle size falls within 26–53 nm as reported for the nanocatalyst in the literature (Veeradate et al., 2012). The SEM image of Fe_2_O_3_ (C) showed flaky crystals with an average size of 6.91 nm (Figure 1E), while that of Fe_2_O_3_ (M) indicated uniformly distributed particles, aggregated together to form a nanocluster with an average particle size of 6.42 nm (Figure 1F). The particle sizes obtained for Fe_2_O_3_ nanoparticles resembled closely those reported by Sweta (2014), but were much lower than the 10 μM reported by Balamurughan et al. (2014). The SEM analysis showed that the microwave synthesized nanoparticles have smaller sizes than that of chemically synthesized.

### UV–Visible and X-Ray Diffraction (XRD) Analyses

The UV absorption of the synthesized metal oxide nanoparticles at the ultraviolet region (not shown) arises from the electronic transition of electrons from the valence band to the conduction band, which might be due to the quantum size particles (Koole et al., 2014). Similar absorption bands has been observed and reported for CuO (Topnani et al., 2010), Al_2_O_3_ (Neethumol et al., 2014), and Fe_2_O_3_ (Balamurughan et al., 2014) nanoparticles.

**Figure 2:**
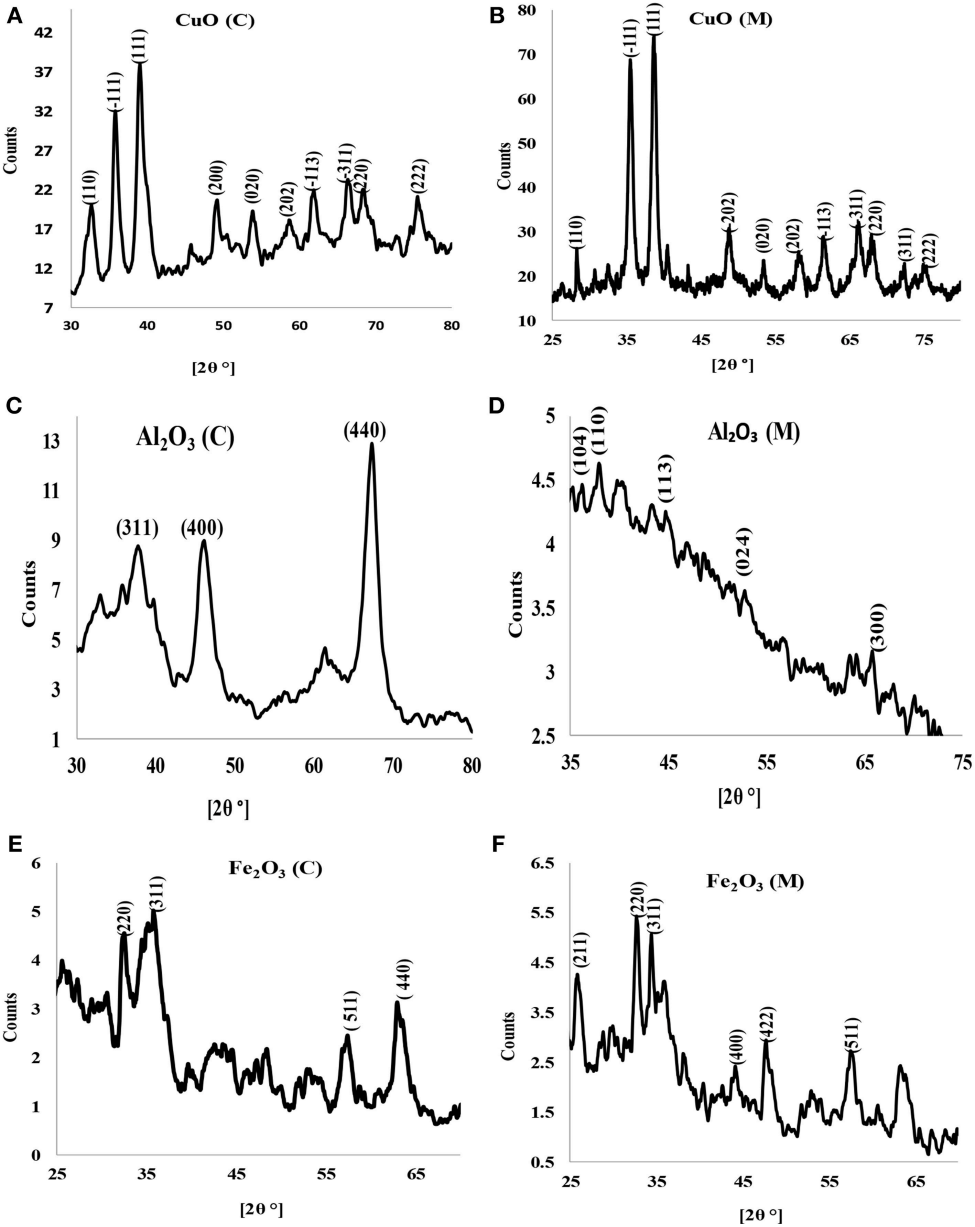
X-ray diffractogram of **(A)** CuO (C) nanoparticles, **(B)** CuO (M) nanoparticles, **(C)** Al_2_O_3_ (C) nanoparticles, **(D)** Al_2_O_3_ (M) nanoparticles, **(E)** Fe_2_O_3_ (C) nanoparticles, and **(F)** Fe_2_O_3_ (M) nanoparticles.

X-ray diffraction is a potent and one of the most important methods for examining the structures of nanomaterials (Takeshi, 2007). X-ray diffractograms of the synthesized nanoparticles are shown in Figures 2A–F. X-ray diffraction pattern of CuO nanoparticles for both chemical and microwave synthesis match with JCPDS 05-0661 library for CuO and is similar to XRD pattern obtained by Dhineshbabu et al. (2016) and Meghana et al. (2015) for CuO nanoparticles. Distinct diffraction peaks at 32.213, 35.497, 38.712, 48.783, 53.551, 61.419, and 66.013 correspond to index planes (110), (−111), (111), (200), (202), (020), (220) (Figure 2A), suggesting a monoclinic structure of CuO (C) nanoparticles (Dhineshbabu et al., 2016). The peaks at 28.234, 35.420, 38.516, 48.650, 57.989, 61.472, 66.020, and 68.056, with index planes at (110), (−111), (200), (−202), (020), (202), (−113), and (311) (Figure 2B) correspond to cubic face CuO (M) nanoparticles (Rejith and Krishnan, 2012). Using Scherrer's formula (Equation 4) below (Neethumol et al., 2014):

(4) $$\begin{array}{r} \text{D}=\frac{\text{Kλ}}{\text{B }\left(\text{Cos }\theta \right)} \end{array}$$

where D is the mean size of crystallites (nm), K is a factor 0.9, λ is x-ray wavelength, B is FWHM and θ is the Bragg angle, the average particle size for CuO (C) and CuO (M) nanoparticles, estimated from full-width at half maximum of the diffraction were 21.75 nm and 21.41 nm, respectively.

X-ray diffractograms of Al_2_O_3_ (C) (Figure 2C) showed broad peaks at 38.421, 45.387, and 67.102, indexes at (311), (400), and (440) plane, correspondent to γ-Al_2_O_3_ phase (according to PDF card No. 00-001-1308) (Abbasian et al., 2010). A weak peak was obtained for Al_2_O_3_ (M) (Figure 2D). The peaks indicate fine crystallinity with an estimated average particle size of 13.05 nm and 9.01 nm for Al_2_O_3_ (C) and Al_2_O_3_ (M), respectively. The XRD pattern for iron oxide nanoparticles for both chemical and microwave synthesis showed that the crystallinity phase is γ-Fe_2_O_3_ (Figures 2E,F). The estimated average particle size for Fe_2_O_3_ (C) particles was 14.11 nm while that of Fe_2_O_3_ (M) was 21.50 nm.

### UV-Vis and FTIR Analysis of Synthesized β-Hematin

In this study, synthetic hemozoin (β-hematin) was used as biomarker instead of the organism (*P. falciparum)* due to the challenges of culturing *P. falciparum* outside the biological system. Hemozoin (β-hematin) is a product of hemoglobin degradation in the bloodstream during infection by the malaria parasite.

**Figure 3:**
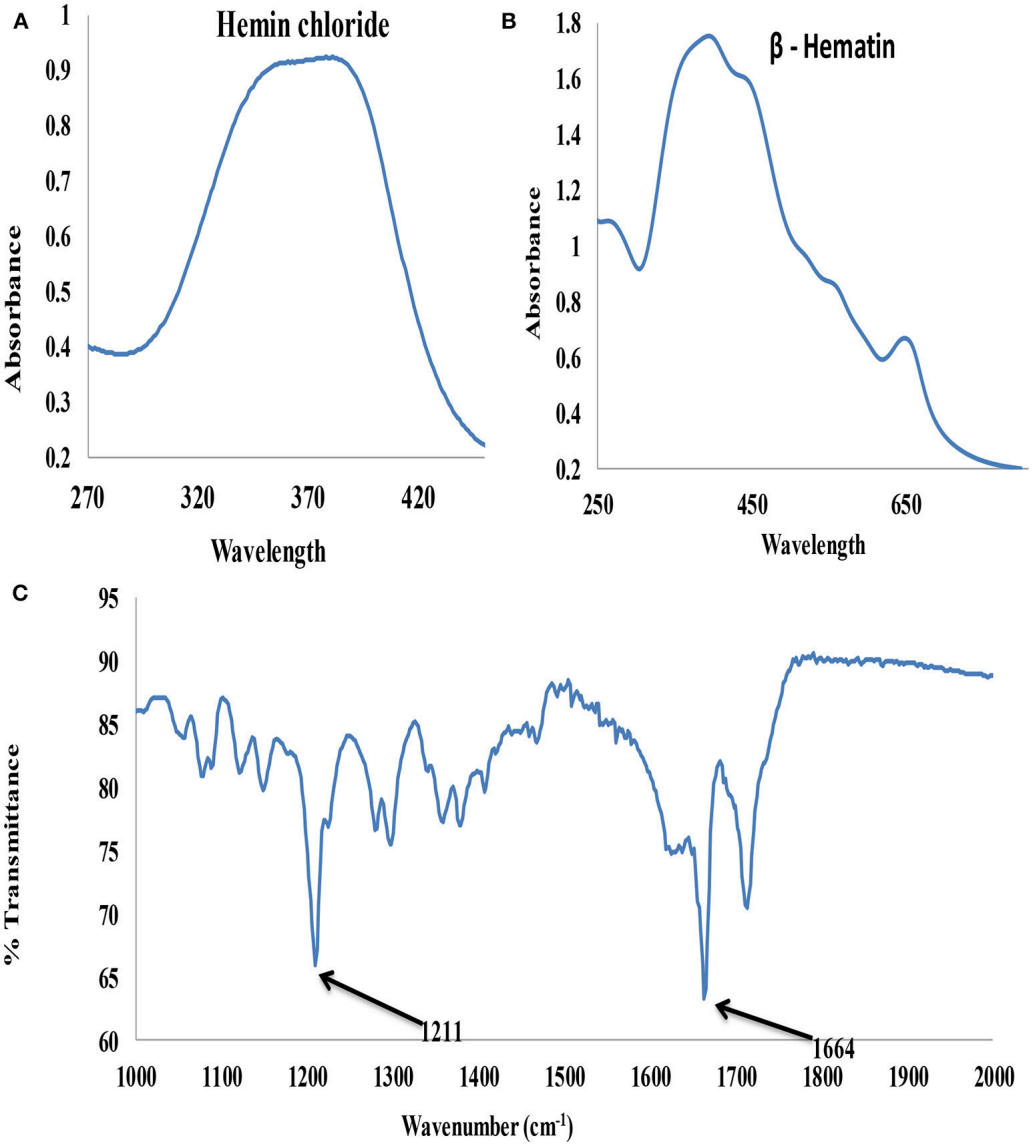
UV–visible spectra **(A)** Hemin chloride **(B)** β-Hematin and **(C)** FTIR spectrum of β-Hematin.

The UV-visible spectroscopy results of hemin chloride (the starting material for β-hematin) and the synthesized β-hematin are showm in Figures 3A,B. The wavelength for maximum absorbance (λmax) for the two molecules is different, confirming the successful conversion of the hemin chloride (Figure 3A) to β-hematin (Figure 3B). Hemin chloride has a broad absorbance at around 370 nm, while the synthesized β-hematin has its characteristic charge transfer (or absorption) band at around 650 nm and two broad soret bands at 383 and 400 nm. The differences in the absorbance of the two materials signify successful synthesis of β-hematin which can be used as a biomarker for the detection of malaria. The UV-visible result of β-hematin obtained in this study is in agreement with that reported by Fitch and Kanjananggulpan (1987) and Laure et al. (2012) for hemozoin or β-hematin.

The infrared spectroscopy analysis further confirmed the successful synthesis of β-hematin with hemozoin characteristic peaks at 1211 and 1664 cm^−1^ (Figure 3C) attributed to a dimeric ferriprotoporphyrin IX aggregate which is identical to *P. falciparum* hemozoin. The intense peak at 1,664 cm^−1^ indicates the presence of C=O stretching, an unidentate carboxylate coordination onto iron in the β-hematin. The peak at 1,211 cm^−1^ indicates an axial carboxylate ligand (C–O stretching frequency) from O-methyl groups linked to various metalloporphyrins (Slater et al., 1991).

### Electrochemical Characterization of Fabricated Nanosensors

**Figure 4:**
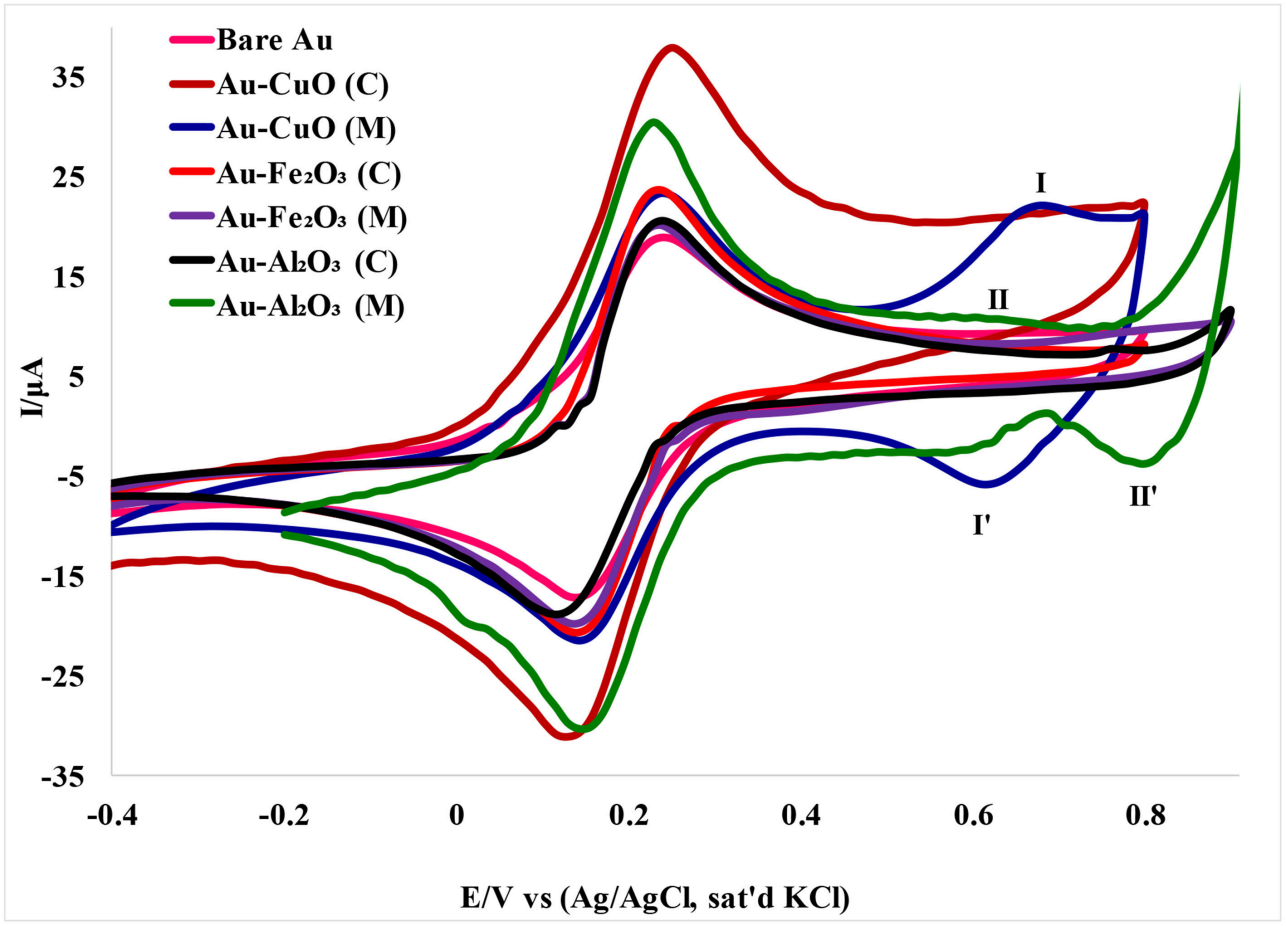
Cyclic voltammograms of Au-MO modified electrodes in 5 mM [Fe(CN)_6_]^4−^/[Fe(CN)_6_]^3−^ redox probe in 0.1 M PBS (scan rate, 50 mV/s).

The electron transport properties of the Au-MO modified electrodes in 5 mM [Fe(CN)_6_]^4−^/[Fe(CN)_6_]^3−^ redox probe in 0.1 M PBS (scan rate, 50 mVs^−1^) were investigated using cyclic voltammetry experiment. Cyclic voltammetric responses of the Au-MO modified electrodes (Figure 4) showed the characteristic one-electron oxidation-reduction peak in the region of 0–0.4 V attributed to [Fe(CN)_6_]^3−/4−^ redox probe but with higher current than that of the bare Au. Also, the characteristic [Fe(CN)_6_]^3−/4−^ redox peaks at the bare Au electrode were similar to those reported by Agboola et al. (2007) for bare Au in [Fe(CN)_6_]^4−^/[Fe(CN)_6_]^3−^ solution.

A pair of redox peaks was also observed for the Au-CuO modified electrode. The redox peaks at regions 0.63 (**I**) and 0.62 V (**I′**) appeared weak for CuO (C) and can be attributed to oxidation of CuO to Cu_2_O_3_ (Zaafarany and Boller, 2009). There were no observable peaks for the Fe_2_O_3_ redox process, probably because of the faster electron transfer process of Fe_2_O_3_ at the electrode, or overlap between the [Fe(CN)_6_]^4−^/[Fe(CN)_6_]^3−^ peaks and the Fe_3_O_4_ redox peaks. However, a peak at 0.80 V (**II′**) was attributed to reduction of Al_2_O_3_ nanoparticles.

The oxidation-reduction peaks observed for Au-Fe_2_O_3_ modified electrode in [Fe(CN)_6_]^4−^/[Fe(CN)_6_]^3−^ solution in this study were in agreement with the oxidation-reduction peaks reported by Fayemi et al. (2018) for Fe_2_O_3_ modified glassy carbon electrode. Electrochemical parameters such as formal potential (E_1/2_), peak-to-peak separation potential (ΔEp), the ratio of anodic and cathodic peak current (I_pa_/I_pc_) were determined and summarized in Table 1. The ratios of the anodic to cathodic peak current (I_pa_/I_pc_) for the bare Au and Au-MO modified electrodes were ~1.0 (unity) which indicates electrochemical reversibility process. Also, all the Au-MO electrodes have lower peak-to-peak separation, ΔEp (80.2–92.6 mV) and thus, enhanced electron transport properties when compared with the bare-Au electrode (ΔEp = 93.6). Factors responsible for enhanced electron transport at the modified electrodes might be the conductive nature of the metal oxide nanoparticle, its facile electronic nature, plus the ionic interaction between the metal oxide nanoparticle and underlying gold (Au) surface. However, Au-CuO (C) and Au-CuO (M) had the lowest ΔEp (80.2 mV) and therefore demonstrated the fastest electron transport among the bare-Au and other Au-MO modified electrodes investigated. It has been reported that the lower the ΔEp, the faster the electron transport at the electrode (Wang, 1994). Although the theoretical peak-to-peak potential separation value (ΔEp) is 59 mV for a fast one-electron process (Wang, 1994), the ΔEp values for all the electrodes were greater than that of the theoretical value.

**Table 1:** Cyclic voltammetric data obtained for the modified electrodes in 5 mM [Fe(CN)_6_]^−3/−4^ in 0.1 M PBS.

**Electrode**	**I_pc_ (μA)**	**I_pa_(μA)**	**I_pa_/I_**pc**_**	**E_pc_ (mV)**	**E_pa_ (mV)**	**E^o'^ (mV)**	**ΔE_p_ (mV)**
Bare Au	17.1	18.8	1.1	143.4	237.0	190.2	93.6
Au-CuO (C)	31.1	37.4	1.2	143.4	223.6	183.5	80.2
Au-CuO (M)	21.5	23.1	1.1	143.4	223.6	183.5	80.2
Au-Fe2O3 (C)	20.7	23	1.1	143.4	236.0	189.7	92.6
Au-Fe2O3 (M)	19.9	19.6	1.0	136.2	223.6	179.9	87.4
Au-Al2O3 (C)	18.8	20.4	1.1	141.6	223.6	182.6	82.0
Au-Al2O3 (M)	30.3	30.5	1.0	142.4	228.8	185.6	86.4

### Electrochemical Catalysis of β-Hematin Using Au-MO Modified Electrodes

**Figure 5:**
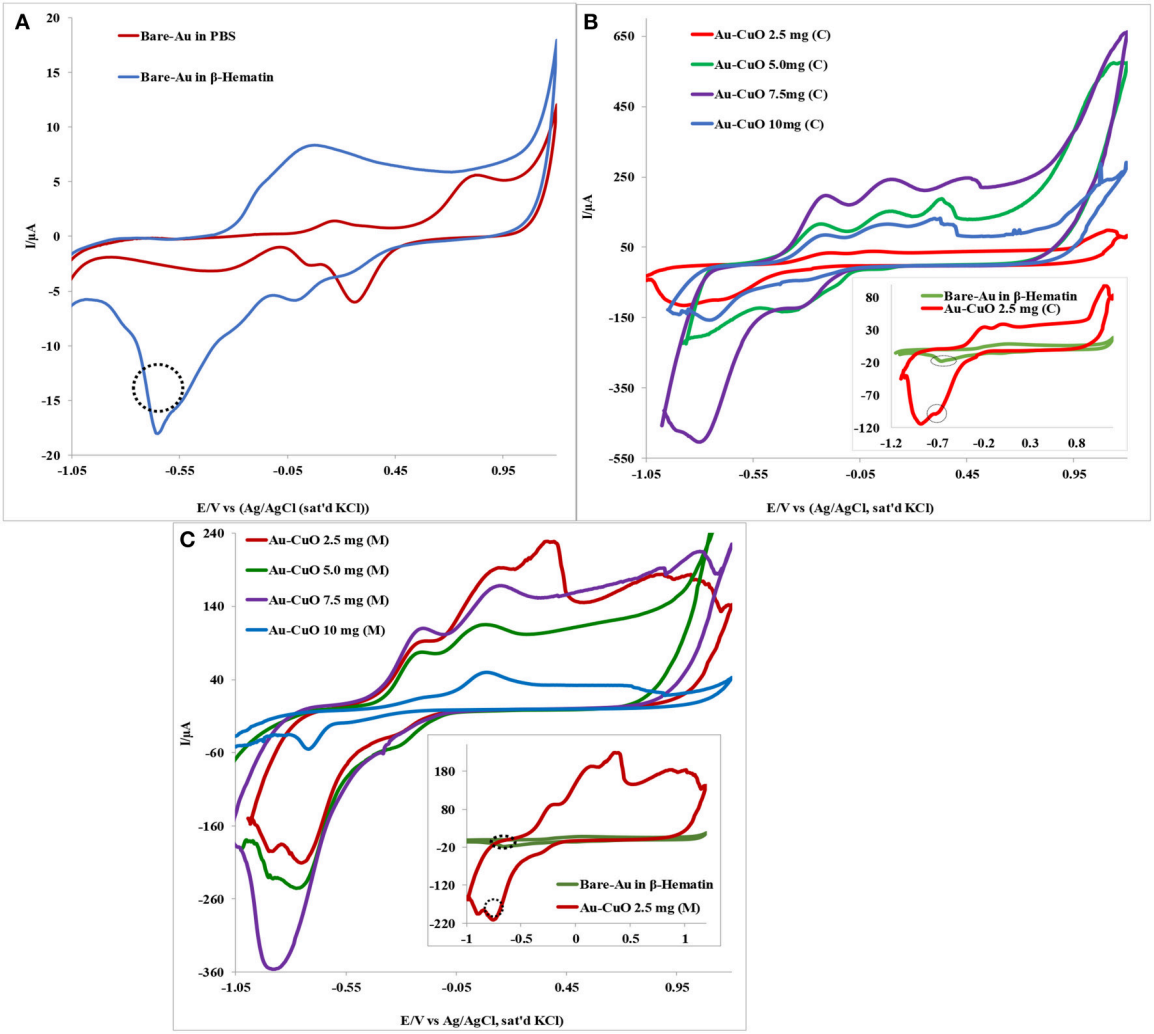
Typical **c**yclic voltammetry evolution of: **(A)** Bare Au in 0.1M PBS (pH 9) and 1.0 mM β-Hematin solution. **(B,C)** Are Au-CuO (C) and Au-CuO (M) (MO weights: 2.5–10.0 mg) modified electrodes in 0.1 M PBS (pH 9) containing 1.0 mM β-Hematin solution.

The electrocatalytic behavior of the Au-MO modified electrode at different weights of MO nanoparticles (2.5, 5.0, 7.5, and 10.0 mg) toward 1.0 mM β-hematin in 0.1 M phosphate buffer solution (pH 9) was investigated using cyclic voltammetric experiment. Figure 5 presents typical voltammogram obtained using Au-CuO modified electrodes. As shown in Figure 5A, the bare Au electrode gave a well-defined β-hematin reduction peak at −0.64 V and current of 17.9 μA not found in PBS (pH 9). However, modifying the Au electrode with 2.5 mg weight of the catalyst, Au-CuO (C) gave β-hematin reduction peak at −0.72 V but at very high current (99.7 μA) compared with bare Au (inset in Figure 5B). Compared with bare Au, the reduction peak of β-hematin at the Au-CuO (C) modified electrode occurred at a lower potential (energy) and higher current response (5 times higher) than those of the bare Au electrode. At 7.5 mg weight of the catalyst, Au-CuO (C) and Au-CuO (M) current responses were observed to be 25 and 18 times higher than that at bare Au (Figures 5B,C).

However, it is interesting to note that the reduction of β-hematin at Au-Fe_2_O_3_ and Au-Al_2_O_3_ modified electrodes still occurred at a lower potential (energy), although at a lower current response compared with the bare Au electrode (not shown). The reason for the low β-Hematin reduction current at Au-Fe_2_O_3_ and Au-Al_2_O_3_ electrodes cannot be established at the moment but could be attributed to the formation of passive layers of Fe_2_O_3_ and Al_2_O_3_ films on Au electrode, thereby hindering electron transport at the electrode after catalysis. The reduction peak observed for β-hematin can be attributed to the reduction of Fe (III) atom at the center of the biomarker which is electrochemically active, to iron (II). Monti et al. (1999) reported the reduction of β-Hematin [(Fe (III) protoporphyrin IX)] to [Fe (II) protoporphyrin IXα] at −0.6 V using a bare platinum electrode as the working electrode, which is in agreement with the −0.64 V reduction potential recorded for β-Hematin at the bare Au electrode in this study. No oxidation peak was observed for β-hematin, suggesting irreversible electrochemical reaction. In general, this result indicates that modifying Au with MO nanoparticles increases its electrode catalysis toward malaria biomarker (β-hematin), with enhanced sensitivity in terms of high reduction current and at lower energy. The remarkable results obtained undoubtedly proved the electrocatalytic sensing of Au-MO modified electrodes toward β-hematin. The results obtained confirmed the potential of the developed sensors for the detection and quantification of β-Hematin (malaria biomarker) or the parasite in biological fluids.

### Scan Rate Study

Cyclic voltammetric experiments were carried out with a view to establishing the effect of scan rate (ν) (scan rates ranging from 25 to 500 mV/s) on the reduction current of β-hematin (1 mM) (Figure S1). It was observed that β-hematin reduction peak increases with an increase in scan rates. Using the Randles-Sevčik equation below, we obtained a linear plot of current (Ip) vs. the square root of scan rate ν^1/2^ (inset in Figure S1).

(5) $$\begin{array}{r} \text{Ip}=\;{2.69\;\times 10}^{5}{\text{n}}^{3/2}{\text{D}}^{\frac{1}{2}}\nu^{\frac{1}{2}}\text{AC} \end{array}$$

The non-zero intercept of the plots suggests that the electrode kinetic process is not completely controlled by the rate of diffusion of β-hematin from the solution to the surface of the electrode. Similar studies in literature have associated such deviation from diffusion to adsorption of reaction intermediates at the electrode surface. Adekunle and Ozoemena (2010) reported adsorption of DEAET intermediate at electrode surface during catalysis of the analytes using SWCNT-Ni modified electrode.

To further confirm the adsorption phenomenon, Tafel value (b) for the electrodes was obtained from plot of (Ep) vs. (log ν) using the Tafel equation (Equation 6) below:

(6) $$\begin{array}{r} \;{\text{E}}_{\text{p}}=\left(\frac{\text{b}}{2}\right)\log\text{ν}+\text{constant} \end{array}$$

Also, from Tafel value b = slope of the Tafel plot, charge transfer coefficient (α) was estimated using the relationship:

(7) $$\begin{array}{r} \text{Slope}=\frac{2.3\text{ RT}}{\text{αnF}} \end{array}$$

where b = Tafel value, R = gas constant, T = temperature (in kelvin), α = charge transfer coefficient, n = number of electron and F = Faraday's constant.

The Tafel values (mVdec^−1^) and the charge transfer coefficient (α) in parenthesis are 1037.2 (0.06), 1042.6 (0.06), 296.2 (0.20), 279.6 (0.21), 174.6 (0.33), 301.8 (0.20) for Au-CuO (C), Au-CuO (M), Au-Fe_2_O_3_ (C), Au-Fe_2_O_3_ (M), Au-Al_2_O_3_ (C), and Au-Al_2_O_3_ (M) electrodes, respectively. The Tafel values are higher than 118.0 mV/dec for a one-electron process (Fayemi et al., 2018). High Tafel value had been attributed to adsorption of reactants or intermediates on the electrode surface (Ju and Leech, 2000). Therefore, it can be inferred from this study that there was adsorption of β-hematin or its intermediate on the surface of the Au-MO electrodes during electrocatalysis. It was also observed that the α values obtained were lower than the value 0.5 for standard reaction mechanism, which indicates that the reaction may be a neutralization reaction, while a value < 0.5 means the reaction is an ionization reaction (Akira et al., 1989). Thus, the lower α values obtained in this study suggest an ionization reaction of the MO and β-hematin. This further confirms the redox process at the electrodes whereby at one point or the other, the MO nanoparticles and the analytes changed from one ionic state to the other with the resultant flow of electrons. The number of electrons (n) involved for each Au-MO electrode was calculated to be one (1). This also suggests that one electron was involved in the reduction of Fe^3+^ at the center of the analyte (β-hematin) by the MO catalyst on Au electrode. Thus, the following mechanism is suggested for the reduction of β-hematin. Assuming the metal ion M^n+^ in MO nanoparticles are represented as Cu^2+^, Fe^3+^, and Al^3+^, respectively, therefore, the proposed mechanism is presented below using M^2+^ as example:

The interaction of the MO nanoparticles with aqueous β-hematin results in the formation of MO-β-hematin adducts (intermediates):

(8) $$\begin{array}{r} {\left(\text{MO}\right)}_{\text{film}}^{2+}+{\text{Fe}}^{3+}\text{PPI}{\text{X}}_{\left(\text{aq}\right)}\to {\left[{\left(\text{MO}\right)}_{\text{film}}^{2+}\ldots \ldots {\text{Fe}}^{3+}\text{PPIX}\right]}_{\text{adduct}} \end{array}$$

The adduct undergoes an internal rearrangement leading to the reduction of Fe^3+^ at the center of β-hematin to Fe^2+^ and oxidation of (MO)^2+^ and (MO)^3+^ simultaneously:

(9) $$\begin{array}{r} {\left[{\left(\text{MO}\right)}_{\text{film}}^{2+}\ldots \ldots {\text{Fe}}^{3+}\text{PPIX}\right]}_{\text{adduct}}\to {\left(\text{MO}\right)}_{\text{film}}^{3+}+{\text{Fe}}^{2+}\text{PPIX}+{\text{e}}^{-} \end{array}$$

Equation 9 represents the one-electron process which is the rate-determining (slow) step. The catalyst is regenerated by the reduction process represented in Equation (10) below:

(10) $$\begin{array}{r} {\left(\text{MO}\right)}_{\text{film}}^{3+}+{\text{e}}^{-}\to {\left(\text{MO}\right)}_{\text{film}}^{2+} \end{array}$$

### Stability Studies

The stability of the Au-MO modified electrodes in β-hematin was confirmed by repetitive cycling of the electrodes in a solution of β-hematin at a scan rate of 50 mV/s. It was observed that the current response at Au-CuO (C) electrode increases by 16.63% after 20 cycles probably due to surface activation (Figure S2A). The electrode was kept in a refrigerator at −4.0°C after use in β-hematin for 2 weeks and run again in the analyte, the current dropped by 14.20% (Inset in Figure S2A). This might be attributed to fouling (or poison) effect of the catalyst at the surface of the electrode after many uses. Similar effects have been noticed and reported for chemically modified electrodes (Ojani et al., 2005). However, the current drop is insignificant and there is assurance of the stability and reusability of the electrode with a reliable result. Other Au-MO electrodes studied showed a drop in β-hematin current after (20 cycles) (Figures S2B–F). The order of the current drop is: Au-Al_2_O_3_ (M) (7.02%) < Au-Al_2_O_3_ (C) (10.61%) < Au-CuO (M) (15.96%) < Au-Fe_2_O_3_ (C) (17.37%) < Au-Fe_2_O_3_ (M) (41.37%) (Figure S2).

In general, except for Au-Fe_2_O_3_ (M) with a high current drop (41.37%), all other developed sensors investigated have demonstrated good stability in the analyte with minimum current drop after 20 cycles. The high decrease in current response observed on Au-Fe_2_O_3_ (M) electrode can be attributed to the poisoning of the electrode surface during the repetitive cycling due to formation of degradation products of β-hematin.

### Electroanalysis Study

The effect of change in concentration of β-hematin on current response at Au-MO modified electrodes was studied using square wave voltammetric (SWV) and chronoamperometric techniques (Figures S3, S4). The square wave analysis was carried out for a concentration range of 1.96–9.91 μM. The result showed that for all the electrodes, current increases with increase in the concentration of β-hematin, 1.96–9.91 μM in PBS (pH 9.0) (Figures S3A–F). The plot of current response (I) vs. β-hematin concentration gave a linear relationship (inset in Figures S4A–F). From these plots, limit of detection (LoD = 3.3 δ/m) and limit of quantification (LoQ = 10 δ/m) where “δ” is the relative standard deviation of the intercept of the y-coordinates from the line of best fit, and “m” the slope of the same line, were determined for the electrodes. The LoD values obtained are 0.83, 0.83, 1.08, 0.77, 0.71, and 0.43 μg/mL for Au-CuO (C), Au-CuO (M), Au-Fe_2_O_3_ (C), Au-Fe_2_O_3_ (M), Au-Al_2_O_3_ (C), and Au-Al_2_O_3_ (M) electrodes, respectively; while the LoQ values are 2.52, 2.52, 3.02, 2.32, 2.15, and 1.30 μg/mL for Au-CuO (C), Au-CuO (M), Au-Fe_2_O_3_ (C), Au-Fe_2_O_3_ (M), Au-Al_2_O_3_ (C), and Au-Al_2_O_3_ (M) electrodes, respectively. It was observed that Au-Al_2_O_3_ (M) has the lowest LoD (0.43 μg/mL) and LoQ (1.30 μg/mL).

The LoDs recorded in this work are higher than 2.5 x 10^−5^ μg/mL and 4.0 x 10^−5^ μg/mL reported for *Plasmodium falciparum histidine-rich protein* 2 (PfHRP 2) detection on screen printed gold electrode (Gikunoo et al., 2014; Aver et al., 2017). Although the LoD is higher for the present study, the use of chemical biomarkers such as β-hematin should be encouraged because they are stable, affordable (cheaper), and easily available than PfHRP 2.

From the chronoamperometry study (Figure S4) and using the Cottrell equation (Bard and Faulkner, 2001), the plots of Ip vs. t^−1/2^ for different concentrations of β-hematin were linear.

(11) $$\begin{array}{r} \text{Ip}=\frac{{\text{nFAD}}^{\frac{1}{2}}\text{C}}{\text{π}{\text{t}}^{\frac{1}{2}}} \end{array}$$

where n is the number of electrons involved in the reaction, F is the Faraday Constant F (96,500 C/mol), and A is the experimentally determined area of the electrode, C is the bulk concentration of the β-hematin (mol/cm^3^), while t is time (s), and D is the diffusion coefficient (cm^2^/s). The diffusion coefficient (D) of β-hematin at the electrode was obtained from the slope of the curve as: Au-CuO (C) (3.5 x 10^−2^ ± 0.0006 cm^2^/s), Au-CuO (M) (1.4 x 10^−2^ ± 0.001 cm^2^/s), Au-Fe_2_O_3_ (C) (3.3 x 10^−2^ ± 0.0004 cm^2^/s), Au-Fe_2_O_3_ (M) (2.4 x 10^−2^ ± 0.0005 cm^2^/s), Au-Al_2_O_3_ (C) (0.5 x 10^−2^ ± 0.0002 cm^2^/s), and Au-Al_2_O_3_ (M) (0.5 x 10^−2^ ± 0.0002 cm^2^/s). The results obtained indicate that diffusion of β-hematin was best favored at Au-CuO (C) electrode and least favored at Au-Al_2_O_3_ electrodes (chemical and microwave synthesized). Also, the Au-MO electrode catalytic rate constants (k) toward β-hematin reduction were estimated from the relationship below (Bard and Faulkner, 2001):

(12) $$\frac{{\text{I}}_{\text{cat}}}{{\text{I}}_{\text{buff}}}=\;\pi^{1/2}{\left(\text{kCt}\right)}^{1/2}$$

where I_catt_ and I_buff_ are the currents of analyte and buffer respectively, C is the analyte bulk concentration, k is the catalytic rate constant and t is the elapsed time. The result obtained follows the order: Au-CuO (C) (1.3 M/s), Au-CuO (M) (1.2 M/s), Au-Al_2_O_3_ (M) (0.6 M/s), Au-Fe_2_O_3_ (M) (0.5 M/s), Au-Al_2_O_3_ (C) (0.5 M/s), and Au-Fe_2_O_3_ (C) (0.3 M/s) The results suggest Au-CuO (C) having better catalytic activity toward the analyte, and that may explain its enhanced performance toward the detection of hematin as demonstrated in this study.

### Interference Study

**Figure 6:**
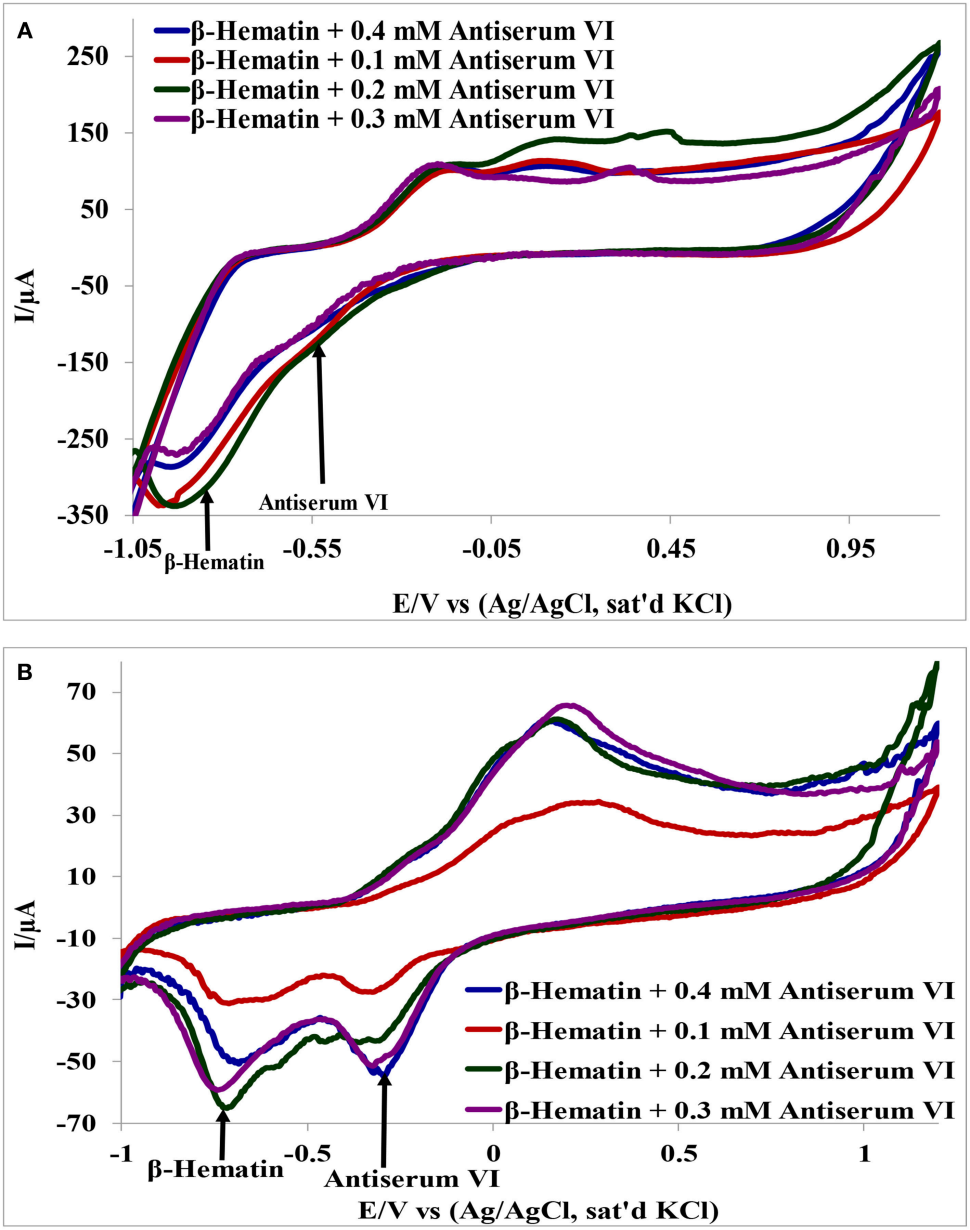
Interference study of **(A)** Au-CuO (C), **(B)** Au-CuO (M) in 1 mM β-Hematin and Different Concentration of Antiserum VI.

Detection of β-hematin on the Au-MO modified electrodes in the presence of *Salmonella typhi* VI antisera was carried out using cyclic voltammetry at a constant concentration of β-hematin (1.0 mM) and varying concentrations of the antiserum. It is interesting to know that β-hematin and antiserum VI could be detected simultaneously with well-defined signal separation of the analytes at their respective potential. A representative cyclic voltammograms using Au-CuO modified electrode is presented in Figure 6. On the other hand, this signal separation was not possible using the bare Au electrode. This result further confirmed the importance of chemically modified electrodes in sensor applications as it enhances the sensitivity and selectivity of bare electrodes. The respective analytes detected potentials and their signal separation at Au-MO electrodes are presented in Table 2. Au-CuO (both chemical and microwave synthesis) gave the highest current response and the best potential separation (500 mV) (Table 2). The result, therefore, suggests that the developed sensors can reliably be used for simultaneous detection of the malaria parasite and typhoid bacteria in clinical samples without signal interferences.

**Table 2:** β-Hematin and *S. typhi* antiserum VI signal potentials at Au-MO modified electrodes and the corresponding signal/peak difference.

**Electrodes**	**β-hematin (mV)**	**Antiserum VI (mV)**	**Peak difference (mV)**
Au-CuO (C)	−910	−410	500
Au-CuO (M)	−900	−400	500
Au-Fe_2_O_3_ (C)	−600	−380	220
Au-Fe_2_O_3_ (M)	−680	−330	350
Au-Al_2_O_3_ (C)	–	–	Broad peak
Au-Al_2_O_3_ (M)	565	375	190

## Analytical Application of the Developed Sensors (Au-MO)

### Recovery Analysis

Since the Au-CuO (C) modified electrode demonstrated superior and impressive electrochemical properties in terms of current response at lower energy with remarkable signal separation of the analytes without interference, its potential for analytical application in real life sample matrices was tested. Au-CuO (C) modified electrode was used for the detection of β-hematin in urine samples (already screened negative) for malaria parasite infection. Five (5) urine samples were spiked with 10 μM of the β-hematin solution and the spiked samples current response at the Au-CuO (C) electrode was determined using square wave voltammetry techniques. It is interesting to know that the β-hematin was successfully detected in the spiked urine samples at around −0.75 V (Figure S5B). The procedure was repeated four (4) times for each urine sample, while β-hematin concentration in the spiked urine samples was determined by extrapolating the current measured from the regression equation of β-hematin standard calibration curve (inset of Figure A). The percentage recovery of the analyte in the urine samples was calculated and presented in Table 3A. The percentage recovery ranged from 90.26 ± 3.22 to 113.58 ± 1.37%, which falls within the recovery range (75–110%) expected for a reliable analytical procedure The result therefore further confirms the suitability and reliability of the Au-MO developed sensors for detection of β-hematin (malaria biomarker) in real life samples.

**Table 3A:** Recovery analysis of β-hematin in human urine samples (*n* = 4).

**Sample**	**Amount added (μM)**	**Amount detected (μM)**	**Recovery (%)**
Urine 1	10.00	11.41 ± 2.19	113.58 ± 1.37
Urine 2	10.00	8.98 ± 1.83	90.26 ± 3.22
Urine 3	10.00	9.27 ± 1.12	93.21 ± 7.22
Urine 4	10.00	9.56 ± 2.48	95.61 ± 4.50
Urine 5	10.00	10.19 ± 2.10	102.38 ± 2.18

### Determination of β-Hematin in Mice and Human Serum

**Figure 7:**
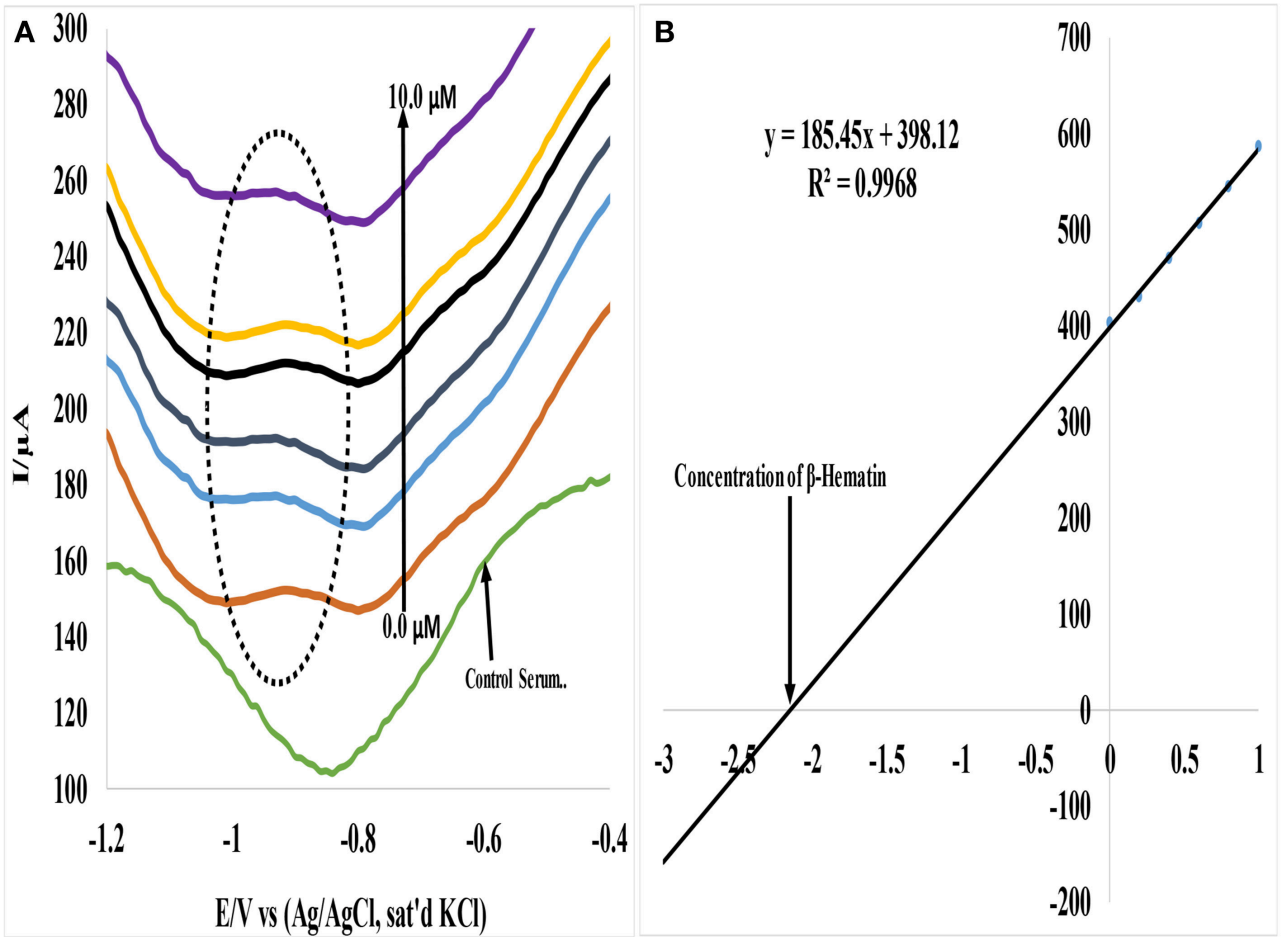
**(A)** Typical SWV analysis of β-hematin in mice serum sample **(B)** Calibration curve for β-hematin determination in unspiked human urine sample using standard addition method (SAM).

In a similar study, Au-CuO (C) modified electrode was used for detection of β-hematin in the serum of infected mice and human sera diagnosed with malaria parasite using square wave voltammetry technique. The procedure followed the standard addition method (SAM). β-hematin peak was observed at around −0.80 V in animal serum and −0.91 V in human serum (Figure 7). This peak was absent in animal serum that was not infected with malaria parasite (control serum, Figure 7A). After spiking the infected serum samples with standard concentrations of β-hematin, the current response increased with increases in concentration (Figure 7A) and the current plot vs. concentration was obtained (Figure 7B). From the plot, the concentrations of β-hematin in the unspiked human and animal serum samples were estimated (Table 3B). The concentrations of β-hematin in the five animal sera analyzed were in the range 3.60 ± 0.33 to 4.80 ± 0.57 mM, while that in human sera were within the range of 0.65 ± 0.02 to 1.35 ± 0.20 mM. The concentrations found in the five human sera samples closely resembled one another and suggest the consistency and reliability of the developed sensor to detect the parasite quantitatively in human serum within the allowable threshold. Although the animal parasite was detected at much higher concentrations in the sera, the trend also suggests the possible threshold within which the parasite can exist in animals.

**Table 3B:** Concentration of β-hematin (mM) in serum samples.

	**Mice serum samples infected with *Plasmodium berghei* (*n* = 4)**	**Human serum samples diagnosed with malaria parasite (*n* = 4)**
**Sample**	**Concentration found (mM)**	**Concentration found (mM)**
Serum 1	4.28 ± 0.11	1.20 ± 0.30
Serum 2	3.75 ± 0.21	0.65 ± 0.020
Serum 3	3.73 ± 0.24	0.88 ± 0.10
Serum 4	4.80 ± 0.57	1.15 ± 0.20
Serum 5	3.60 ± 0.33	1.35 ± 0.20

Cyclic voltammetry technique was also used to confirm the presence of β-hematin in human serum using Au-CuO (C) electrode and the reduction peak was successfully observed at around −0.82 V (inset in Figure S6). Infected animal serum was also spiked separately with each of *S. typhi* antiserum VI for simultaneous detection in the presence of the malaria biomarker (β-hematin) (Figure S6). The developed sensor successfully separated β-hematin from the antiserum with well-defined signal separation of 250 mV (Figure S6). The results also confirmed the potential of the Au-MO developed sensors as a device for detection and quantification of the malaria parasite and typhoid bacteria in human serum sample with little or no diagnosis confusion of malaria and a different antiserum.

## Conclusion

This study describes the electron transport and electrocatalytic properties of both chemically and microwave synthesized metal oxide nanoparticles (MO) (where M = Cu, Fe, and Al) deposited on gold electrode toward the detection of malaria biomarker (β-Hematin) in clinical samples (e.g., blood serum, urine etc.). The study demonstrated that electrocatalysis of β-hematin was more favored on Au-electrode modified with CuO nanoparticles (chemical and microwave synthesized). The outstanding properties of the Au-CuO electrodes compared to others include enhanced current response at lower onset potential for catalysis, high electrochemical stability in the analytes (resistance to electrode fouling) and good signal separation between malaria and typhoid biomarkers (i.e., little or no interference) during detection. Au-CuO (C) gave the best sensing properties toward the detection of β-hematin in urine and blood serum. The sensor showed well-defined peaks of β-hematin and *S. typhi* antiserum VI in the clinical samples and was able to detect both analytes simultaneously with a potential separation of 250 mV in serum.

## Author Contributions

ASA and JAO designed the work and were part of the manuscript write-up, TS, TTIN and BBM provided the research platform for nanoparticles characterization, ORO carried out the experiment, interpreted the results and prepared the manuscripts. All authors reviewed the manuscript and agreed to its publication.

### Conflict of Interest Statement

The authors declare that the research was conducted in the absence of any commercial or financial relationships that could be construed as a potential conflict of interest.
