# Supplementary material for: Development of Electrochemical Nanosensor for the Detection of Malaria Parasite in Clinical Samples
Source: Front Chem. 2019 Feb 25;7:89. doi: 10.3389/fchem.2019.00089 (PMC6397833; doi:10.3389/fchem.2019.00089)
Supplement: Supplementary file 1 [file Data_Sheet_1.docx]

**(a)**

**(b)**

**(c)**

**(d)**

**(e)**

**(f)**

**Figure S1**: Scan Rate Study of (a) Au-CuO (C), (b) Au-CuO (M), (c) Au-Fe_2_O_3_ (C), (d) Au-Fe_2_O_3_ (M), (e) Au-Al_2_O_3_ (C) and (f) Au-Al_2_O_3_ (M) at 25 – 500 mV/s in 0.1 M PBS containing 1mM β-Hematin

**(b)**

**(a)**

**(d)**

**(c)**

**(e)**

**(f)**

**Figure S2:** Cyclic Voltammograms (20 cycles) showing the **s**tability of: (a) Au-CuO (C), (b) Au-CuO (M), (c) Au-Fe_2_O_3_ (C), (d) Au-Fe_2_O_3_ (M), (e) Au-Al_2_O_3_ (C) and (f) Au-Al_2_O_3_ (M) electrodes in 0.1 M PBS containing 1.0 mM β-Hematin (scan rate: 50 mV/s).

**(a)**

**(b)**

**(c)**

**(d)**

**(f)**

**(e)**

**Figure S3:** Concentration Study of: (a) Au-CuO (C), (b) Au-CuO (M), (c) Au-Fe_2_O_3_ (C), (d) Au-Fe_2_O_3_ (M), (e) Au-Al_2_O_3_ (C) and (f) Au-Al_2_O_3_ (M) Electrodes in 0.1 M PBS containing 1mM β-Hematin using Square Wave Voltammetry Technique.

**(f)**

**(e)**

**(d)**

**(c)**

**(b)**

**(a)**

**Figure S4:** Plot of peak current (I_p_) versus t^-½^ for: (a) Au-CuO (C) (b) Au-CuO (M), (c) Au-Fe_2_O_3_ (C), (d) Au-Fe_2_O_3_ (M), (e) Au-Al_2_O_3_ (C) and (f) Au-Al_2_O_3_ (M) Electrodes in 0.1 M PBS containing 1mM β-Hematin using Chronoamperometric Technique.

**(a)**

**(b)**

**Figure S5:** (a) Calibration Curve for β-Hematin Standards (2, 4, 6, 8, 10 μM) (b) Square Wave Voltammograms of urine samples (n=5) spiked with 10 μM β-Hematin using Au-CuO(C) electrode.

**Figure S6:** Typical cyclic voltammograms showing the detection of β-Hematin in an infected mouse in the presence and varying concentrations of antiserum VI. (Inset: overlaid CVs confirming the presence of β-hematin in human serum (at around -0.82 V) and -0.7 V for antiserum VI in human serum using Au-CuO (C) electrode; while the “Control” is the CV for uninfected human serum sample respectively).
